# The Emerging Role of Neurosteroids: Novel Drugs Brexanalone, Sepranolone, Zuranolone, and Ganaxolone in Mood and Neurological Disorders

**DOI:** 10.7759/cureus.65866

**Published:** 2024-07-31

**Authors:** Malay Singhal, Nishi Modi, Lajpat Bansal, Jeby Abraham, Ishani Mehta, Arun Ravi

**Affiliations:** 1 Neurology, Mahatma Gandhi Memorial Medical College, Indore, IND; 2 Psychiatry and Behavioral Sciences, Government Medical College Surat, Surat, IND; 3 Psychiatry and Behavioral Sciences, Maharaja Agrasen Institute of Medical Research and Education, Hissar, IND; 4 General Medicine, Yenepoya Medical College, Mangalore, IND; 5 Neurology, Maharaja Agrasen Institute of Medical Research and Education, Hissar, IND; 6 Psychiatry and Behavioral Sciences, Sri Ramachandra Institute of Higher Education and Research, Chennai, IND

**Keywords:** parkinson's disease, epilepsy, post-partum depression, mood disorders, ganaxolone, zuranolone, sepranolone, brexanolone, neurosteroids

## Abstract

This review investigates the potential of neurosteroids, including brexanolone, zuranolone, sepranolone, and ganaxalone, as therapeutic agents for a range of mood and neurological disorders. Notably, these disorders encompass postpartum depression, post-traumatic stress disorder (PTSD), major depressive disorder (MDD), epilepsy, and Alzheimer's disease.

Brexanolone and zuranolone have emerged as frontrunners in the treatment of postpartum depression, offering rapid relief from debilitating symptoms. Their mechanism of action involves modulation of the gamma-aminobutyric acid (GABA) system, which plays a pivotal role in mood regulation. Clinical trials have demonstrated their efficacy, heralding a potential breakthrough in addressing this often-overlooked condition. In the context of PTSD and MDD, neurosteroids have demonstrated significant promise. Their positive allosteric modulation of GABA-A receptors translates into improved mood stabilization and reduced symptoms. This novel approach represents a departure from conventional treatments and could offer newfound hope for individuals grappling with these disorders.

Beyond mood disorders, neurosteroids, especially ganaxalone, exhibit potential in the realm of epilepsy management. Ganaxalone's capacity to control seizures is attributed to its GABAergic activity, which helps restore the delicate balance of neurotransmission in epileptic brains. Moreover, neurosteroids have revealed neuroprotective properties in Alzheimer's disease models. By influencing the GABAergic system, they mitigate excitotoxicity, a hallmark of Alzheimer's pathology. This neuroprotection opens a novel avenue for slowing neurodegeneration, although further research and clinical validation are essential.

In conclusion, this review underscores the substantial therapeutic promise of neurosteroids in mood and neurological disorders. Their modulation of the GABA system emerges as a central mechanism of action, emphasizing the importance of GABAergic signaling in these conditions. The path forward entails continued investigation and clinical trials to fully unlock the potential of neurosteroids, offering hope for enhanced treatments in these challenging clinical domains.

## Introduction and background

Neurosteroids are a distinct class of steroids synthesized within the brain, which play a crucial role in influencing diverse neurophysiological functions such as myelination, neuronal development, and neurotransmission. Unlike traditional steroid hormones, neurosteroids have a unique mechanism of action as they interact with intracellular receptors, and directly affect ligand-gated ion channels or membrane receptors. This distinctive characteristic allows neurosteroids to swiftly modulate neuronal signaling, making them a subject of interest and investigation [1]. Neurosteroids are capable of binding to and interacting with receptors such as gamma-aminobutyric acid-A (GABA-A), N-methyl-D-aspartate (NMDA), glycine, and opioid σ1 receptors [2]. This involvement extends to essential physiological effects, including neuronal plasticity, learning and memory functions, aggression, and epilepsy. Additionally, they also influence responses to stress, anxiety, and depression [3]. Several studies have studied the role of neurosteroids in neuroinflammation and their subsequent contribution to neurodegenerative disorders such as multiple sclerosis (MS), Alzheimer's disease (AD), and Parkinson's disease (PD). Endogenous neurosteroids are broadly classified into three categories: pregnane, androstane, and sulfated [4]. Our study delves into their, current knowledge of biosynthesis, intricate mechanisms, functional roles, and pathophysiological implications, shedding light on their potential therapeutic significance.

Types of neurosteroids

Pregnane Neurosteroids: Allopregnanolone and Allotetrahydrodeoxycorticosterone (THDOC)

They are neuroactive compounds. Progesterone is produced by neurons and glial cells in the nervous system. Enzymes necessary for converting cholesterol to pregnenolone and pregnenolone to progesterone are widely distributed in the brain. Furthermore, it can be metabolized to generate neuroactive metabolites, with allopregnanolone being the most crucial one [5]. Allopregnanolone and pregnanolone have the ability to influence neuronal activity by enhancing GABAergic neurotransmission. This enhancement is achieved through their potent and positive allosteric modulation of GABA action at GABA-A receptors. Notably, when administered before stress, allopregnanolone reduces the endocrine response to stress. This effect is likely due to the inhibition of CRH neurons through GABA-mediated mechanisms. The action seems to be linked to the tonic activation of extrasynaptic GABA-A receptors that contain the δ subunit, rendering them particularly sensitive to the effects of neurosteroids [6].

Androstane Neurosteroids: Androstanediol, Etiocholanone

Dehydroepiandrosterone (DHEA) may have a promising function in alleviating stress and fostering resilience in humans [7]. The rise in serum DHEA levels showed a connection with reduced activity in both the amygdala, an essential area for emotional significance, and the hippocampus, which plays a role in contextual memory and fear response. Consequently, DHEA diminishes emotional reactivity by regulating negative emotions. Additionally, DHEA increases activity in the rostral anterior cingulate cortex (rACC), potentially enhancing cognitive regulation and suppressing negative emotional reactivity. Given that these abilities are compromised in certain anxiety disorders, DHEA acts as an anxiolytic agent by influencing GABA-A receptors [8].

Sulfated Neurosteroids: Pregnenolone Sulfate (PS), Dehydroepiandrosterone Sulfate (DHEAS)

Sulfated neurosteroids PS and DHEAS, along with DHEA, exhibit evident antidepressant effects in both animals and humans. During pregnancy, there is a significant increase in neurosteroid levels derived from progesterone, but these levels rapidly decline after delivery. Given that neuroactive steroids possess anxiolytic properties and the withdrawal of neurosteroids leads to heightened anxiety behavior, it is plausible that neurosteroids may play a crucial role in the development of postpartum depression [9].

Mechanism of action

Neurosteroids and GABA

Neurosteroids, similar to other steroid hormones, can influence gene transcription by interacting with nuclear receptors. Additionally, they have the ability to interact with plasma membrane G protein-coupled receptors, thereby affecting the binding of neuropeptides to their receptors. Neurosteroids also demonstrate the capacity to stimulate tubulin polymerization in cultured neurons by binding to the microtubule-associated protein-2. However, the majority of neurosteroid actions are primarily carried out through allosteric modulation of neurotransmitter receptors, specifically the GABA-A/central-type benzodiazepine receptor (CBR) complex illustrating its significance in mediating their actions [10]. Studies have demonstrated that Neurosteroids act on a wide range of GABA-A receptor subtypes present in the brain. However, there appears to be a preference for binding with extrasynaptic receptors containing the delta subunit [11].

Neurosteroids are among the most potent and efficacious modulators currently identified for GABA receptors. To contextualize this, let us compare neurosteroids with well-known GABA receptor modulators such as benzodiazepines and barbiturates. Neurosteroids demonstrate potency equivalent to benzodiazepines, yet they exhibit significantly higher efficacy. On the other hand, barbiturates are as efficacious as neurosteroids, but they notably fall short in terms of potency when compared to neurosteroids [12]

Most neurosteroids potentiate the response of GABA-A receptors at low GABA concentrations. Some neurosteroids, mostly sulfated ones, can also inhibit the response of GABA-A receptors at all concentrations of GABA. The inhibition or potentiation is thought to be mediated by different binding sites on receptors [13]. At lower concentrations, it is thought that neurosteroids potentiate the response to subsaturating levels of GABA at the GABA-A receptor. When average ambient GABA levels are ≤1 μm, the potentiation seems to play a major role in neuronal excitability. But at higher concentrations, neurosteroids directly open the chloride-gated channels on GABA-A receptors. The significance of direct gating in relation to the cellular and behavioral effects of neuroactive steroids is often deemed negligible. This conclusion is based on the general observation that substantially higher concentrations, surpassing those required for anesthesia, are necessary to activate the channels. However, a study by Shu et al. showed that even small currents generated by direct gating of GABA channels can have significant effects on membrane excitability [14].

Effect of Various Neurotransmitters on Neurosteroids

Neurotransmitters significantly influence the synthesis and activity of neurosteroids, which are crucial for various physiological functions. The interactions between these chemical messengers and neurosteroids can affect mood, cognition, and behavior. Table 1 summarizes the effects of different neurotransmitters on neurosteroid production and modulation.

**Table 1:** Effect of various neurotransmitters on neurosteroids. GABA, gamma-aminobutyric acid; DHEA, dehydroepiandrosterone, DHEAS, dehydroepiandrosterone sulfate. [10]

Neurotransmitter	Effect on neurosteroids	Potential mechanism
Glutamate	Inhibitory	Rapid inhibitory effect of α-amino -3-hydroxy-5-m ethyl-4-isoxazo lepropionic acid (AMPA) or N-methyl-D-aspartate (NMDA) on P450 aromatase activity can be ascribed to Ca2+-dependent phosphorylation of the P450 aromatase protein
Melatonin	Mirrors the variations in plasma melatonin concentrations	Melatonin inhibits the expression of CYP7B and that subsequently decreases 7α-OH-Δ5P in the brain and causes nocturnal reduction of locomotor activity.
Prolactin (PRL)	Stimulatory	PRL directly acts on Mg neurons expressing CYP7B to enhance the biosynthesis of 7α-OH-Δ5P, which in turn mediates the stimulatory effect of PRL on locomotion.
GABA	GABA has been shown to inhibit in a dose-dependent manner de novo biosynthesis of various neurosteroids including 17OH-Δ5P, P, 17OH-P, and DHEA	GABA inhibits the biosynthesis of neurosteroids through activation of GABA-A receptors,
Endozepine	Stimulatory	Endozepines have been shown to regulate steroid secretion by adrenocortical cells and Leydig cells.
Vasotocin and mesotocin	Stimulatory	Vasotocin acting through a V1a-like receptor, and mesotocin acting through an MT receptor, stimulate the biosynthesis of 17OH-Δ5P, DHEA, 17OH-P, and P.
Neuropeptide-Y	Inhibitory	Neuropeptide-Y acting through Y1 receptors negatively coupled to adenylyl cyclase, inhibits the biosynthesis ofΔ5PS and DHEAS

Physiology of GABA

Since the predominant effect of neurosteroids on GABA, it becomes imperative to understand the physiology of GABA and its role in various disorders.

GABA/GABA-A receptor signaling is the primary inhibitory pathway in the central nervous system (CNS). This inhibition occurs in two forms: phasic and tonic inhibition. Phasic inhibition involves transient stimulation of GABA-A receptors by GABA, leading to a decrease in postsynaptic neuron excitability. On the other hand, tonic inhibition is considered a continuous mechanism of inhibition that regulates excitation through long-term hyperpolarization. Tonic inhibition plays a vital role in synaptic plasticity, neurogenesis, and cognitive functions. Any disruption in either phasic or tonic inhibition is linked to various neurological and psychiatric disorders. Consequently, modulating these signals has become the foundation of drug therapy and anesthesia [15].

Mood disorders: In mood disorders, the mature GABAergic system undergoes changes during late puberty, transitioning from excitatory neurotrophic actions to inhibitory responses, and ensuring the proper functioning of receptor kinetics. The cognitive and emotional aspects of mood disorders are closely linked to the GABAergic neurocircuitry, particularly in mesocorticolimbic regions like the ventral tegmental area (VTA), substantia nigra (SN), nucleus accumbens (NAc), basolateral amygdala, hippocampus, and medial prefrontal cortex. Other regions involved include the subthalamic nucleus, habenula, dorsal raphe nucleus, anterior cingulate cortex, and cerebellum. In depressive disorders, the neural circuits responsible for slow and fast neuroadaptation may be disrupted due to altered G protein coupling efficacy or/and endocytosis of the membrane-delimited receptors [16]. These changes contribute to the pathophysiology of mood disorders, affecting the balance of inhibitory responses and leading to dysregulated neurocircuitry. In preclinical models of despair and major depressive disorder, canonical signal transduction cascades are involved. When monoamine neurotransmitters bind to their specific receptors, G protein adapters are recruited to the receptors' intracellular C-terminal tail. This activation of G proteins can either stimulate (Gs) or inhibit (Gi) the cAMP/PKA/CREB cascade. Adenylyl cyclase converts adenosine triphosphate (ATP) into cyclic adenosine monophosphate (cAMP), which then activates protein kinase A (PKA), leading to the phosphorylation of cAMP-response element binding protein (CREB). Phosphorylated CREB translocates to the nucleus, where it stimulates the transcription of target genes involved in neuroprotection, neurotransmission, and cytoskeletal dynamics. Neurotrophins, on the other hand, bind to their specific receptor tyrosine kinases, resulting in the autophosphorylation of their intracellular domains. This autophosphorylation process recruits various adapter proteins to the plasma membrane, initiating protein-protein interactions that eventually activate Raf, a protein kinase. Raf further activates the small molecule Ras, leading to the induction of mitogen-activated protein kinase (MAPK). Similar to the cAMP/PKA/CREB cascade, the MAPK/ERK pathway results in the translocation of transcription factors (including CREB) to the nucleus [17]. These signaling cascades play significant roles in the neurobiological mechanisms underlying despair and major depressive disorder.

Epilepsy: Changes in the expression and function of GABA-A receptors have been observed and are believed to be involved in the development of epilepsy. There are different types of GABA-A receptors in the brain; synaptic receptors contain γ subunits, while those located at perisynaptic or extrasynaptic sites predominantly contain δ subunits. Electrophysiologically, these receptors are responsible for phasic and tonic inhibition. During status epilepticus (SE), a state of prolonged seizures, there is increased neuronal hyperexcitability, compromising inhibitory GABAergic synaptic transmission. This results in reduced miniature inhibitory postsynaptic currents (mIPSCs) and decreased active GABA-A receptors per dentate granule cell [18]. In the epileptic brain, an overall increase in the mean frequency of neuronal discharges and a rise in the number of bursting neurons was observed. Interestingly, the inhibitory effect of exogenously applied GABA on neuronal activity was significantly enhanced, whereas the efficacy of GABA-A and GABA-B receptor blockers decreased, suggesting changes in intraseptal inhibitory processes in epilepsy. Furthermore, in epilepsy, GABA has a pronounced effect of increasing the oscillatory activity of a specific group of pacemakers, while the opposite effect is observed in the control group. Additionally, in epileptic animals, GABA receptor blockers did not influence burst neurons, indicating a disruption of the tonic GABAergic control over the oscillatory activity [19]. These findings shed light on the alterations in GABAergic regulation in epileptic conditions and their impact on neural activity in the septal region of the brain.

Alzheimer's disease: The loss of local inhibitory GABAergic networks in Alzheimer's disease contributes to excitotoxicity due to excessive influx of Ca2+, resulting in hyperphosphorylation of tau protein, leading to the formation and aggregation of neurofibrillary tangles [20].

## Review

Brexanolone

In a USA-based double-blind placebo control trial, female inpatients with severe post-partum depression (PPD) (Hamilton Rating Scale for Depression {HAM-D} total score ≥26) were randomly assigned to receive a 60-hour continuous intravenous (IV) dose of either brexanolone or placebo [21]. A total of 21 women were randomly assigned. The infusion of brexanolone in women experiencing severe PPD led to a notable and significant decrease in the HAM-D total score, demonstrating clinical relevance when compared to the placebo. After 60 hours, the brexanolone group showed a mean reduction of 21.0 points (SE 2.9) in the HAM-D total score from baseline, while the placebo group had a mean reduction of 8.8 points (SE 2.8). Adverse events were reported in four out of 10 patients in the brexanolone group and eight out of 11 patients in the placebo group, with dizziness and somnolence being the most frequently reported adverse events in the brexanolone group. These findings provide support for the approach of targeting synaptic and extrasynaptic GABA-A receptors in the development of treatments for individuals with PPD [21]. According to the study by Epperson et al., based on the outcomes from the HUMMINGBIRD program, which comprised one Phase 2 and two Phase 3 double-blind, randomized, controlled trials, brexanolone stands as the sole treatment approved for PPD in the United States [22]. These post hoc analyses included adults with PPD who were randomly assigned to receive either a 60-hour infusion of brexanolone 90 μg/kg/h or placebo across three trials. Patients treated with Brexanolone90 achieved a more rapid response in HAMD-17 and Clinical Global Impression of Improvement (CGI-I), with higher response rates compared to the placebo group. Additionally, improvements in HAMD-17 Anxiety/Somatization and Insomnia subscales favored Brexanolone90, starting at 24 hours and continuing through day 30. In women with PPD, brexanolone demonstrated rapid improvement in depressive symptoms as well as symptoms of anxiety and insomnia when compared to placebo. These findings further reinforce the effectiveness of brexanolone as a treatment option for adults with PPD [22].

In trials conducted by Cooper et al., brexanolone injection demonstrated a significantly greater reduction in the HAM-D total score compared to placebo [23]. The results of the efficacy data were also compared with those of selective serotonin reuptake inhibitors (SSRIs), the most commonly prescribed class of antidepressants for PPD using the HAM-D and Edinburgh Postnatal Depression Scale (EPDS). The results from multiple treatment comparisons (MAICs) consistently showed larger improvements in change from baseline for brexanolone compared to SSRIs across various time points. Differences in change from baseline for the HAM-D between brexanolone and SSRIs were significant, with larger improvements observed on day 3, week 4, and the last observation. Similarly, for the EPDS, brexanolone exhibited significant differences in change from baseline, indicating greater improvements on day 3, week 4, and the last observation. These findings highlight the importance of employing the Multiple Treatment Comparisons method to account for heterogeneity in placebo arms and provide additional evidence supporting the efficacy of brexanolone compared to SSRIs [23]. Based on preclinical evidence conducted by Kanes et al., rapid fluctuations in allopregnanolone levels, a metabolite of progesterone, may contribute to PPD [24]. Brexanolone (formerly known as SAGE-547 Injection), an injectable formulation of allopregnanolone was assessed in an open-label, proof-of-concept study as a treatment for severe PPD. Four women diagnosed with severe PPD, characterized by a baseline HAM-D score of ≥20, were treated with brexanolone. The dosage of brexanolone administered was adjusted to replicate allopregnanolone levels typically observed in the third trimester of pregnancy. A 36-hour maintenance infusion was followed by a 12-hour tapering period. The primary focus of the study was on safety measures, while secondary assessments included evaluating efficacy using measures such as the HAM-D. From the initial measurement after the start of infusion until hour 84, there was a consistent decrease in mean HAM-D total scores, indicating a reduction in symptoms consistent with remission. Additionally, all other assessments of efficacy demonstrated similar improvements [24].

In the study conducted by Wald et al., 12 participants received a 60-hour infusion of brexanolone with a titration up to 90 µg/kg/h [25]. The concentrations of allopregnanolone were measured in both breast milk and plasma. The results showed that allopregnanolone concentration-time profiles were similar between breast milk and plasma, with a partition coefficient of 1.36. The peak concentration (Cmax) in plasma was 89.7 ng/mL, and the median time to reach maximum concentration (Tmax) was 47.8 hours. There were no notable differences in the area under the concentration-time curve (AUC) and Cmax between participants with or without concomitant antidepressant treatment. The maximum relative infant dose (RID) was 1.3%. The rapid elimination of allopregnanolone from plasma and breast milk, along with the low relative infant dose, supports the appropriate use of weight-based dosing for brexanolone and aligns with other pharmacokinetic-related labeling recommendations [25].

Zuranolone

The in vitro pharmacological characteristics of zuranolone were investigated using various experimental approaches. Heterologous expression of recombinant human GABA-A receptors in human embryonic kidney (HEK) cells and Xenopus oocytes showed that zuranolone acts as a positive allosteric modulator (PAM) at multiple GABA-A receptor subunit combinations, enhancing the potency and efficacy of GABA-induced currents. In addition, zuranolone exhibited synergistic effects with the benzodiazepine diazepam, indicating cooperative interactions at the receptor level. Acute treatment with zuranolone in mouse hippocampal slices resulted in increased tonic GABA currents and prolonged spontaneous inhibitory post-synaptic currents (sIPSCs). In vivo studies demonstrated that zuranolone had favorable pharmacokinetic properties, with high oral bioavailability and brain penetration. It also showed pharmacodynamic activity, as it increased the latency to tonic seizures in a mouse model and modulated EEG activity in rats. These findings support the potential therapeutic use of zuranolone as a novel pharmacological treatment targeting GABA receptors [26]. 

In a phase 3 trial involving 151 adult women suffering from PPD, a double-blind, randomized, placebo-controlled approach was employed which suggested the potential for the short-term, outpatient utility of zuranolone. The participants who received 30 mg zuranolone daily for two weeks experienced significantly greater reductions in depressive symptoms compared to those who received placebo by the 15th day as measured on HAMD-17 score (−17.8 vs −13.6; difference, −4.2). These reductions in depressive symptoms were noticeable as early as the third day (difference, −2.7) and remained consistent at all the assessed time intervals until the 45th day (difference, −4.1). The study found sustained differences in favor of zuranolone at day 15. These differences were observed in HAMD-17 response (odds ratio was 2.63), HAMD-17 score remission (odds ratio was 2.53), change from baseline for Montgomery-Åsberg Depression Rating Scale (MADRS) score (difference, -4.6), and Hamilton Rating Scale for Anxiety score (difference, -3.9) [27]. The phase 3 MOUNTAIN study aimed to evaluate the effectiveness of zuranolone 20 mg and 30 mg in MDD. However, it did not achieve its primary objective of demonstrating a significant improvement in depressive symptoms compared to placebo at day 15, as measured by the Hamilton Depression Rating Scale (HDRS-17) total score. However, the study results suggest that zuranolone may offer a novel approach for treating MDD. Patients receiving zuranolone 30 mg experienced a rapid onset of symptom improvement as early as day 3 of the 14-day treatment course. Although the response rates in patients receiving zuranolone 30 mg were not significantly different from the placebo group, there were similar response rates between day 15 and day 182. This indicates that the therapeutic effects of zuranolone persisted beyond the treatment period [28]. 

Results from a phase 2 clinical trial assessing the impact of zuranolone on health-related quality of life (HRQoL) in individuals with MDD demonstrated significant improvements. The trial was a randomized, double-blind study involving a total of 89 participants, with 45 receiving zuranolone 30 mg and 44 receiving a placebo. After a 15-day treatment period, patients who received zuranolone exhibited notable enhancements in various domains of the Short Form-36v2 Health Survey (SF-36v2), a widely used HRQoL assessment tool. The improvements surpassed the thresholds for minimally important differences in multiple scales, including Bodily Pain, General Health, Vitality, Social Functioning, Role Emotional, and Mental Health. The observed changes in these scales ranged from 3 to 7.8 points, indicating clinically significant improvements. Comparatively, patients receiving zuranolone demonstrated statistically significant improvements in General Health, Vitality, Mental Health, and Mental Component Summary when compared to the placebo group (p ≤ 0.025) [29]. The phase 3 ROBIN study randomized oral zuranolone 30 mg or placebo once daily for 14 days The study showed that women treated with zuranolone achieved concurrent improvements in both depressive and anxiety symptoms as early as day 3. A greater proportion of women receiving zuranolone achieved a sustained response on both days 15 and 45, compared to those on placebo (59% vs. 39%, NNT {number needed to treat} = 5). Similarly, sustained remission rates were also higher with zuranolone (37% vs. 13%, NNT = 5). Regarding safety and tolerability, the NNH (number needed to harm) estimates for discontinuation due to adverse events (AEs) and experiencing ≥1 treatment-emergent AE (TEAE) were both ≥10, indicating a favorable safety profile for zuranolone. The incidence of AEs ≥2% was higher with zuranolone compared to placebo, but the NNH estimates for specific AEs were not provided due to the small sample size. The study's results suggest that zuranolone may offer a potential rapid-acting pharmacotherapy for adults with PPD, including those experiencing anxiety and/or insomnia symptoms, potentially without the need for polypharmacy [30]. A trial comprising 89 patients with a diagnosis of moderate-to-severe MDD based on the 17-item HAM-D score of 22 or higher were randomized in a 1:1 ratio to receive either zuranolone 30 mg or placebo for 14 days. Results showed that patients who received SAGE-217 experienced a greater reduction in depressive symptoms compared to those who received placebo, with a least-squares mean change in HAM-D score of -17.4 points for SAGE-217 and -10.3 points for placebo (p < 0.001). Moreover, at day 15, 79% of patients in the SAGE-217 group achieved a reduction of more than 50% from baseline in the HAM-D score, compared to only 41% in the placebo group. No serious adverse events or deaths were reported during the trial. Although the trial had limitations, such as a small sample size and lack of adjustment for multiplicity in secondary endpoint analyses, this phase 2 trial suggests that SAGE-217 may be a promising treatment option for MDD [31]. Another study enrolled 45 participants, randomized into different treatment groups. Both the 30 mg and 45 mg doses of zuranolone significantly improved objective measures of sleep efficiency (SE), duration, and maintenance compared to the placebo (p < 0.001 for both doses). Additionally, subjective measures of sleep quality (SQ) were significantly improved with zuranolone compared to placebo (p < 0.001). Zuranolone also increased the time spent in Stage N2 and N3 non-REM sleep stages, which contributed to the overall increase in total sleep time (TST). There were no significant next-day effects on sleepiness or psychomotor performance, but some participants in the 45 mg group reported increased sleepiness. The study demonstrated that zuranolone has the potential as a treatment for insomnia and may be particularly relevant for individuals with comorbid major depressive disorder (MDD) and insomnia. Zuranolone's unique pharmacology as a GABA-A receptor-positive allosteric modulator of both synaptic and extrasynaptic receptors differentiates it from other insomnia pharmacotherapies, providing a novel treatment approach [32].

Sepranolone

During a phase 2 double-blinded, 206 females diagnosed with premenstrual dysphoric disorder (PMDD) were treated with sepreanolone 10 mg and 16 mg. The treatment effect was assessed across six parameters. Summarized 21 symptom scores during the third treatment cycle showed significant improvement in symptom scores during nine premenstrual days for the 10 mg sepranolone group, demonstrating a statistically significant difference compared to placebo (p = 0.027). Treatment effect on impairment scores after three cycles demonstrated that 54.5% of participants in the placebo group, 80% in the 10 mg group, and 75.5% in the 16 mg group no longer experienced impairment in work performance. Only the 10 mg sepranolone group exhibited a significant treatment effect on PMDD distress compared to placebo. Neither the 16 mg treatment group nor the combined active treatment groups (10 mg and 16 mg) showed a statistically superior effect to placebo (p = 0.184) while comparing the treatment effect on change in Sum21 (the score for all 21 symptom questions in the DRSP) PMDD symptoms. A responder analysis for treatment cycle 3, focused on achieving symptom-free or minimal symptoms on the Daily Record of Severity of Problems (DRSP) scale (<30/42), demonstrated the superiority of the 10 mg sepranolone treatment. The results indicate a positive signal that during ovulatory luteal phases in women with PMDD, the 10 mg sepranolone dosage may alleviate negative mood symptoms, improve distress, and reduce impairment to a greater extent than the placebo [33].

In a multi-center study conducted in Sweden, researchers carried out an exploratory investigation to assess the pharmacokinetic parameters of sepranolone administered via subcutaneous injection [34]. The study was divided into two phases: a pharmacokinetic phase I study involving 26 healthy women and a phase II study with 126 women diagnosed with PMDD. The phase II study revealed promising results for sepranolone as a potential treatment for PMDD, as it led to a substantial 75% reduction in total DRSP scores, compared to a 47% reduction observed in the placebo group. The effect size of sepranolone was found to be similar to that of widely used treatments for PMDD, such as SSRIs and oral contraceptives containing drospirenone. The overall symptom improvement achievable with sepranolone therapy was estimated to be a reduction of -45.5 scale steps on the DRSP. The actual treatment effect of sepranolone showed a reduction of -34.4 (-75%), while the placebo exhibited a reduction of -21.5 (-47%). This substantial 75% symptom reduction corresponds to an improvement from severe to mild/minimal on the DRSP scale. The effect size, ranging from 0.64 to 0.73, demonstrated consistency across total DRSP score, negative mood, and impairment scores, aligning with findings from previous studies on SSRIs and drospirenone-containing oral contraceptives used to treat PMDD [34].

Ganaxolone

In a pilot study, the effects of ganaxalone (positive allosteric modulator of GABA-A receptor) as an adjunctive therapy for depression in post-menopausal women despite ample treatment with antidepressants were investigated. The trial consisted of 10 post-menopausal women with persistent depression despite ample antidepressant therapy for more than six weeks. Ganaxolone 225 mg twice daily or 450 mg twice daily for eight weeks followed by a two-week taper showed a significant decrease in MADRS score (24.4 ± 1.6 to 12.8 ± 2.9). More than a 50% decrease in MADRS score was observed in 44% (4/9) participants which persisted even after a two-week taper period. Additionally, there were improvements in MADRS reduced sleep subscore, depressive symptomatology-self report score, and subscales for disruption in sleep quality. 100% of participants experienced sleepiness and fatigue out of which 60% also experienced dizziness [35]. In another trial, ganaxolone was tested as a treatment for Post-traumatic stress disorder (PTSD) in 112 participants. The dosing was 200 mg, increased to 400 mg, and further increased to 600 mg twice daily two times a week. It was found that the expected therapeutic blood level of ganaxolone was not evident in over 35% of the participants who received ganaxolone at the conclusion of the double-blind phase. However, ganaxolone didn't show significant improvement compared to placebo. The low blood levels of ganaxolone in some participants may have contributed to the lack of efficacy. Future studies should consider higher dosing and targeted treatment for specific PTSD subpopulations [36]. 

In a phase 2 trial, ganaxolone showed promise as an effective monotherapy for patients with refractory status epilepticus. Researchers evaluated the efficacy of IV ganaxolone in patients with refractory status epilepticus who had not responded to benzodiazepines and at least one IV antiseizure medication. The administration of IV ganaxolone included an initial bolus followed by a continuous infusion with decreasing rates for 48-96 hours, followed by an 18-hour taper period. The dosing was divided into three cohorts: low (500 mg/day), medium (650 mg/day), and high (713 mg/day). The median time for cessation of status epilepticus episodes after ganaxolone infusion initiation was five minutes. Notably, none of the patients required a third-line IV anesthetic during the 24-hour period after the ganaxolone infusion [37]. Another clinical trial conducted to assess the safety and antiepileptic activity of ganaxolone showed positive results in 52 patients who were withdrawn from their epileptic medication. The first group of 24 patients received ganaxolone at a dose of 1500 mg/d on day 1 and 1875 mg/d on days 2 to 8. The second group, comprising 28 patients, received a placebo. The completion rate was higher in the ganaxolone group (50%) compared to the placebo group (25%). Covariate analyses showed a significant treatment effect on survival time in men. Post-hoc chi2 probe analyses focusing on participants who completed the entire study revealed a notable difference between the treatment groups. Adverse effects were reported by 79% of participants treated with ganaxolone, while 68% of participants in the placebo group reported side effects [38]. An open-label pilot study evaluated the efficacy of ganaxolone in pediatric and adolescent populations, aged 5-15 years with refractory epilepsy. They received ganaxolone with beta-cyclodextrin (1:1 ratio) in an oral suspension. The dosage was gradually increased from 1 mg/kg, b.i.d. to 12 mg/kg t.i.d. over 16 days. The study showed that four out of 15 participants experienced a 50% decrease in seizure frequency, while two showed a 25-50% decrease. The remaining participants were considered non-responders with less than a 24% reduction in seizure frequency. Somnolence was reported as a side effect by nine participants [39].

In a phase 2 clinical study aimed to assess the efficacy of ganaxolone in Protocadherin 19 (PCDH19) clustering epilepsy enrolled 21 females aged 1-17 years with molecularly confirmed PCDH19 variants, experiencing at least 12 seizures during the 12-week screening period. Patients were randomized to receive either ganaxolone (n = 10) or placebo (n = 11) in addition to their standard antiseizure treatment for a 17-week double-blind phase. Ganaxolone demonstrated a greater reduction in seizure frequency (-61.5%) compared to placebo (-24.0%) after the 17 weeks, but the difference did not reach statistical significance (p = 0.17). Ganaxolone was generally well-tolerated, with somnolence being the most common TEAE, although serious TEAEs were more prevalent in the placebo group [40].

Although these initial findings are promising, it is important to note that these treatment options have not been extensively studied, and the clinical trials conducted thus far have been relatively small. As such, more comprehensive and larger-scale research is needed to fully understand the efficacy, safety, and long-term impact of these neurosteroids. Further studies will be crucial in establishing robust clinical guidelines and ensuring that patients receive the most effective and safe treatments based on a thorough understanding of this novel pharmacological approach.

Neurosteroids and current standard treatment

Neurosteroids, such as brexanolone, zuranolone, sepranolone, and ganaxolone, treat mood and neurological disorders by primarily modulating GABA-A receptors [41]. Unlike SSRIs, which focus on serotonin reuptake inhibition, neurosteroids enhance GABAergic transmission. This mechanism allows neurosteroids to reduce mood symptoms within hours to days, compared to the weeks or months typically required for SSRIs to take effect. This rapid onset is particularly helpful in situations needing quick relief, such as severe postpartum depression or acute mood disorder exacerbations [42]

Despite their benefits, neurosteroids come with certain limitations. Clinical trials have commonly reported drowsiness, dizziness, and fatigue [43]. While these effects are generally manageable, they need to be carefully considered, particularly for individuals who may experience sedation or cognitive impairment. Additionally, there are concerns about the potential for abuse or dependence on GABA-modulating drugs, making close monitoring and regulation crucial.

On the other hand, neurosteroids may offer a valuable alternative for patients who cannot tolerate the side effects of SSRIs. SSRIs are known to cause issues like sexual dysfunction, weight gain, and gastrointestinal problems, which often lead to discontinuation of the medication. Given their different side effect profiles, neurosteroids could provide a more tolerable option for some individuals [44].

## Conclusions

In conclusion, the literature review on neurosteroids and their efficacy in mood disorders reveals promising findings across various clinical settings. The four neurosteroids studied have shown positive outcomes in patients with conditions such as postpartum depression, post-traumatic stress disorder, and major depressive disorder. These results suggest that neurosteroids could represent a novel and effective treatment approach for mood disorders, offering hope for patients who may not have responded adequately to traditional therapies. They may also provide the benefit of more rapid symptom relief compared to conventional treatments, making them especially valuable in acute care settings. Emerging research on ganaxolone's potential in treating epilepsy and related syndromes shows promise as an area of investigation. Initial studies have reported positive outcomes, yet more extensive research with larger sample sizes and clearly defined indications is necessary to confirm its efficacy. While the early findings are encouraging, it is crucial to interpret these results with caution and continue rigorous scientific exploration to establish the role of ganaxolone in epilepsy management fully. The use of neurosteroids in treating mood disorders and epilepsy presents a significant potential breakthrough, providing new therapeutic options for individuals with these conditions. Further research and clinical trials are essential to deepen our understanding of neurosteroids' safety, efficacy, and optimal use across different patient populations.

## References

[1] Sze Y, Brunton PJ (2022). Neurosteroids and early-life programming: an updated perspective. Curr Opin Endocr Metab Res.

[2] Biagini G, Panuccio G, Avoli M (2010). Neurosteroids and epilepsy. Curr Opin Neurol.

[3] Maurice T, Phan VL, Urani A, Kamei H, Noda Y, Nabeshima T (1999). Neuroactive neurosteroids as endogenous effectors for the sigma1 (sigma1) receptor: pharmacological evidence and therapeutic opportunities. Jpn J Pharmacol.

[4] Reddy DS (2010). Neurosteroids: endogenous role in the human brain and therapeutic potentials. Prog Brain Res.

[5] Yilmaz C, Karali K, Fodelianaki G, Gravanis A, Chavakis T, Charalampopoulos I, Alexaki VI (2019). Neurosteroids as regulators of neuroinflammation. Front Neuroendocrinol.

[6] Henderson VW (2018). Progesterone and human cognition. Climacteric.

[7] Almeida FB, Pinna G, Barros HM (2021). The role of HPA axis and allopregnanolone on the neurobiology of major depressive disorders and PTSD. Int J Mol Sci.

[8] Ben Dor R, Marx CE, Shampine LJ, Rubinow DR, Schmidt PJ (2015). DHEA metabolism to the neurosteroid androsterone: a possible mechanism of DHEA's antidepressant action. Psychopharmacology (Berl).

[9] Sripada RK, Marx CE, King AP, Rajaram N, Garfinkel SN, Abelson JL, Liberzon I (2013). DHEA enhances emotion regulation neurocircuits and modulates memory for emotional stimuli. Neuropsychopharmacology.

[10] Vaudry H, Ubuka T, Soma KK, Tsutsui K (2022). Editorial: recent progress and perspectives in neurosteroid research. Front Endocrinol (Lausanne).

[11] Stell BM, Brickley SG, Tang CY, Farrant M, Mody I (2003). Neuroactive steroids reduce neuronal excitability by selectively enhancing tonic inhibition mediated by delta subunit-containing GABAA receptors. Proc Natl Acad Sci U S A.

[12] Akk G, Covey DF, Evers AS, Steinbach JH, Zorumski CF, Mennerick S (2007). Mechanisms of neurosteroid interactions with GABA(A) receptors. Pharmacol Ther.

[13] Akk G, Bracamontes JR, Covey DF, Evers A, Dao T, Steinbach JH (2004). Neuroactive steroids have multiple actions to potentiate GABAA receptors. J Physiol.

[14] Shu HJ, Eisenman LN, Jinadasa D, Covey DF, Zorumski CF, Mennerick S (2004). Slow actions of neuroactive steroids at GABAA receptors. J Neurosci.

[15] Ghit A, Assal D, Al-Shami AS, Hussein DE (2021). GABA(A) receptors: structure, function, pharmacology, and related disorders. J Genet Eng Biotechnol.

[16] Wang YS, Qiu TY, Fu Q (2022). Unravelling biological roles and mechanisms of GABA(B)R on addiction and depression through mood and memory disorders. Biomed Pharmacother.

[17] Niciu MJ, Ionescu DF, Mathews DC, Richards EM, Zarate CA Jr (2013). Second messenger/signal transduction pathways in major mood disorders: moving from membrane to mechanism of action, part I: major depressive disorder. CNS Spectr.

[18] González MI, Brooks-Kayal A (2011). Altered GABA(A) receptor expression during epileptogenesis. Neurosci Lett.

[19] Malkov AE, Popova IY (2011). Functional changes in the septal GABAergic system of animals with a model of temporal lobe epilepsy. Gen Physiol Biophys.

[20] Ali AB, Islam A, Constanti A (2023). The fate of interneurons, GABA(A) receptor sub-types and perineuronal nets in Alzheimer's disease. Brain Pathol.

[21] Kanes S, Colquhoun H, Gunduz-Bruce H (2017). Brexanolone (SAGE-547 injection) in post-partum depression: a randomised controlled trial. Lancet.

[22] Epperson CN, Rubinow DR, Meltzer-Brody S (2023). Effect of brexanolone on depressive symptoms, anxiety, and insomnia in women with postpartum depression: Pooled analyses from 3 double-blind, randomized, placebo-controlled clinical trials in the HUMMINGBIRD clinical program. J Affect Disord.

[23] Cooper MC, Kilvert HS, Hodgkins P, Roskell NS, Eldar-Lissai A (2019). Using matching-adjusted indirect comparisons and network meta-analyses to compare efficacy of brexanolone injection with selective serotonin reuptake inhibitors for treating postpartum depression. CNS Drugs.

[24] Kanes SJ, Colquhoun H, Doherty J, Raines S, Hoffmann E, Rubinow DR, Meltzer-Brody S (2017). Open-label, proof-of-concept study of brexanolone in the treatment of severe postpartum depression. Hum Psychopharmacol.

[25] Wald J, Henningsson A, Hanze E, Hoffmann E, Li H, Colquhoun H, Deligiannidis KM (2022). Allopregnanolone concentrations in breast milk and plasma from healthy volunteers receiving brexanolone injection, with population pharmacokinetic modeling of potential relative infant dose. Clin Pharmacokinet.

[26] Althaus AL, Ackley MA, Belfort GM (2020). Preclinical characterization of zuranolone (SAGE-217), a selective neuroactive steroid GABA(A) receptor positive allosteric modulator. Neuropharmacology.

[27] Deligiannidis KM, Meltzer-Brody S, Gunduz-Bruce H (2021). Effect of zuranolone vs placebo in postpartum depression: a randomized clinical trial. JAMA Psychiatry.

[28] Clayton AH, Lasser R, Nandy I, Sankoh AJ, Jonas J, Kanes SJ (2023). Zuranolone in major depressive disorder: results from MOUNTAIN-A phase 3, multicenter, double-blind, randomized, placebo-controlled trial. J Clin Psychiatry.

[29] Suthoff E, Kosinski M, Arnaud A (2022). Patient-reported health-related quality of life from a randomized, placebo-controlled phase 2 trial of zuranolone in adults with major depressive disorder. J Affect Disord.

[30] Deligiannidis KM, Citrome L, Huang MY (2023). Effect of zuranolone on concurrent anxiety and insomnia symptoms in women with postpartum depression. J Clin Psychiatry.

[31] Gunduz-Bruce H, Silber C, Kaul I (2019). Trial of SAGE-217 in patients with major depressive disorder. N Engl J Med.

[32] Bullock A, Gunduz-Bruce H, Zammit GK (2022). A phase 1 double-blind, placebo-controlled study of zuranolone (SAGE-217) in a phase advance model of insomnia in healthy adults. Hum Psychopharmacol.

[33] Bäckström T, Ekberg K, Hirschberg AL (2021). A randomized, double-blind study on efficacy and safety of sepranolone in premenstrual dysphoric disorder. Psychoneuroendocrinology.

[34] Bixo M, Ekberg K, Poromaa IS (2017). Treatment of premenstrual dysphoric disorder with the GABA(A) receptor modulating steroid antagonist Sepranolone (UC1010)-A randomized controlled trial. Psychoneuroendocrinology.

[35] Dichtel LE, Nyer M, Dording C (2020). Effects of open-label, adjunctive ganaxolone on persistent depression despite adequate antidepressant treatment in postmenopausal women: a pilot study. J Clin Psychiatry.

[36] Rasmusson AM, Marx CE, Jain S (2017). A randomized controlled trial of ganaxolone in posttraumatic stress disorder. Psychopharmacology (Berl).

[37] Vaitkevicius H, Ramsay RE, Swisher CB, Husain AM, Aimetti A, Gasior M (2022). Intravenous ganaxolone for the treatment of refractory status epilepticus: results from an open-label, dose-finding, phase 2 trial. Epilepsia.

[38] Laxer K, Blum D, Abou-Khalil BW, Morrell MJ, Lee DA, Data JL, Monaghan EP (2000). Assessment of ganaxolone's anticonvulsant activity using a randomized, double-blind, presurgical trial design. Ganaxolone Presurgical Study Group. Epilepsia.

[39] Pieribone VA, Tsai J, Soufflet C, Rey E, Shaw K, Giller E, Dulac O (2007). Clinical evaluation of ganaxolone in pediatric and adolescent patients with refractory epilepsy. Epilepsia.

[40] Sullivan J, Gunning B, Zafar M (2023). Phase 2, placebo-controlled clinical study of oral ganaxolone in PCDH19-clustering epilepsy. Epilepsy Res.

[41] Cutler AJ, Mattingly GW, Maletic V (2023). Understanding the mechanism of action and clinical effects of neuroactive steroids and GABAergic compounds in major depressive disorder. Transl Psychiatry.

[42] Hutcherson TC, Cieri-Hutcherson NE, Gosciak MF (2020). Brexanolone for postpartum depression. Am J Health Syst Pharm.

[43] Patatanian E, Nguyen DR (2022). Brexanolone: a novel drug for the treatment of postpartum depression. J Pharm Pract.

[44] Giatti S, Diviccaro S, Cioffi L, Falvo E, Caruso D, Melcangi RC (2021). Effects of paroxetine treatment and its withdrawal on neurosteroidogenesis. Psychoneuroendocrinology.

